# An Unlikely Culprit: Novel Case of Vancomycin Oral Solution-Induced Agranulocytosis

**DOI:** 10.7759/cureus.103484

**Published:** 2026-02-12

**Authors:** Ryan Azarkhail, Uchemdi Nduka, Haaroon Tariq, Prasad Rao

**Affiliations:** 1 Internal Medicine, Wellstar Kennestone Hospital, Marietta, USA; 2 Internal Medicine, Ross University School of Medicine, Bridgetown, BRB

**Keywords:** agranulocytosis, c diff, clostridium difficile infection treatment, diarrhea due to c diff, drug induced agranulocytosis, fidaxomicin, oral vancomycin, vancomycin-induced agranulocytosis

## Abstract

Vancomycin is a glycopeptide antibiotic commonly used for the treatment of *Clostridioides difficile *colitis and other severe Gram-positive infections. While hematologic toxicities such as neutropenia and agranulocytosis are rare, they are typically associated with intravenous administration. We report the first known case of agranulocytosis secondary to oral vancomycin solution. A 44-year-old African American woman with a history of hypertension and prior alcohol use presented with severe diarrhea, palpitations, and hypokalemia after completing six days of oral vancomycin tablets. On admission, she was transitioned to vancomycin oral solution 125 mg four times daily. Her absolute neutrophil count (ANC) was 1.76 × 10⁹/L on admission and declined to 1.03 by hospital day 5. Extensive workup excluded nutritional deficiencies, viral infections, and concomitant medications as alternative causes. Colonoscopy demonstrated erythematous mucosa and chronic inflammation in the sigmoid colon, suggesting increased systemic absorption of oral vancomycin. The drug was discontinued and replaced with fidaxomicin, leading to normalization of ANC and resolution of diarrhea. This case underscores the importance of vigilance for hematologic complications even with oral vancomycin, particularly in patients with intestinal inflammation. Early recognition, discontinuation of vancomycin, and substitution with alternative therapy are key to recovery.

## Introduction

Discovered in 1952, vancomycin is a tricyclic glycopeptide antibiotic that functions by blocking the processes of transpeptidation (cross-linking of NAM pentapeptide chains) and transglycosylation (addition of precursors to the nascent peptidoglycan chain), both of which are essential for the formation and maintenance of a stable cell wall [1-3]. Increased osmotic pressure and ultimately cell lysis result from disruption of cell wall integrity, especially in Gram-positive bacteria [1].

Oral vancomycin is regarded as the main treatment for intestinal Clostridium difficile infection (CDI) [4]. For initial, recurrent, and fulminant CDI, it is advised to be used as a first-line treatment [5-7]. Additionally, C. difficile-induced gastrointestinal infections, pseudomembranous colitis, and staphylococcal enterocolitis can all be treated with oral vancomycin [2]. According to the Infectious Diseases Society of America (IDSA) guidelines, oral vancomycin is the first-line treatment for fulminant CDI, while vancomycin or fidaxomicin is the recommended antibiotic of choice for an initial episode of CDI [6]. Fidaxomicin is a macrocyclic antibiotic with a restricted spectrum and minimal systemic absorption, rendering it a preferred alternative for patients who develop intolerance or adverse effects to vancomycin. Data suggest that fidaxomicin provides superior clinical benefits as well as reduced rates of recurrence; however, its use is limited due to cost and accessibility [8-10].

Vancomycin can be formulated for intravenous (IV) or oral administration; intravenous dosage is determined by clinical presentation, kidney function, body weight, and serum concentrations, necessitating close monitoring of drug levels and kidney function, especially in patients with impaired renal function [2]. Oral vancomycin, on the other hand, is minimally absorbed by an intact gastrointestinal tract, leading to high intraluminal concentrations, which can be beneficial against C. difficile [2]. Its systemic bioavailability is considered negligible, generally below 10%, meaning routine therapeutic monitoring of vancomycin levels is not typically recommended, and dosage adjustment for renal impairment is usually unnecessary [2, 11].

We present the first reported instance of agranulocytosis as sequelae to consumption of oral vancomycin solution. Our case is particularly unique, since agranulocytosis induced by IV vancomycin is already a rare adverse effect, and would be less likely in oral formulations. However, our case suggests that sufficient systemic exposure can occur, especially in the context of disrupted gastrointestinal mucosa due to colitis. Mucosal inflammation may facilitate absorption, increasing the risk of systemic side effects traditionally associated with intravenous use.

## Case presentation

A 44-year-old African-American female with a history of hypertension and occasional alcohol use presented with palpitations, nausea, vomiting, and profuse diarrhea. She denied smoking, recent alcohol use, radiation exposure, inflammatory bowel disease, or dietary triggers. She was admitted for electrolyte management due to significant hypokalemia of 2.6 mmol/L.

Initial CT abdomen and pelvis with IV contrast was unremarkable for acute infectious or inflammatory process (see Videos 1-2).

**Video 1:** CT Abdomen/Pelvis with IV Contrast (Coronal View)

**Video 2:** CT Abdomen/Pelvis with IV Contrast (Axial View)

Physical examination revealed only mild diffuse abdominal tenderness. Initial laboratory workup was notable for hypokalemia, hypomagnesemia, presence of white blood cells (WBC) in stool, and C. Diff Carrier state (see Table 1).

**Table 1:** Initial Laboratory Workup mmol = millimole; L = Liter; mg = milligram; dL = deciliter; mm = millimeters; hr = hour; pg = picograms; mL = milliliter; min = minute; U = Unit; WBC = White Blood Cells Labs performed on admission.

Lab Test	Patient Result	Reference Range / Interpretation
Potassium	2.6 mmol/L	3.5 - 5.1 mmol/L
Magnesium	1.0 mg/dl	1.6 - 2.6 mg/dL
Hepatitis A/B/C serology	Non-reactive	Non-reactive indicates no detectable antigen/antibody
Legionella urine antigen	Negative	Negative = normal
CRP	<0.3 mg/dL	<0.5 mg/dL
ESR	<1 mm/hr	0–19 mm/hr
Ethanol level	<10 mg/dL	Undetectable in non-users
Vitamin B12	305	232 - 1,245 pg/mL
Folate	9	>4.7 ng/mL
Creatinine	0.40 mg/dL	0.5–0.9 mg/dL
GFR	126 mL/min	>59 ml/min/1.73 m^2^
Gliadin IgA	3.3 U/mL	<15.0 indicates antibody not detected
Transglutaminase IgA	<1.0 U/mL	<15.0 indicates antibody not detected
WBC stool	Positive	Negative = no inflammation in GI tract
Clostridium difficile Toxin A/B, PCR	Detected	Positive C. Diff PCR and Negative C Diff Toxin most commonly represents a carrier state
C. difficile Toxin Test	Negative	

Five days prior to admission, she had tested positive for C. difficile via PCR (toxin negative) and was prescribed oral vancomycin 125 mg four times per day (QID). Despite reported compliance, she continued to experience 10-20 watery, mucousy bowel movements daily.

Upon admission, vancomycin tablets were switched to oral vancomycin solution 125 mg QID. Her Absolute Neutrophil Count (ANC) on admission was 1.76 (see Table 2). While receiving the oral vancomycin solution from hospital days 1 to 5, her ANC declined steadily (see Table 3).

**Table 2:** ANC/Clinical Findings

Lab Test	Patient Result	Reference Range / Interpretation
Absolute Neutrophil Count (ANC)	1.76 x 10^9^/L	1.70 - 7.00 x 10^9^/L
White Blood Cell (WBC) Count	3.37 x 10^9^/L	3.50 - 10.50 x 10^9^/L
Absolute Reticulocyte Count	114.7 x 10^9^/L	29.5 - 87.3 x 10^9^/L
%Retics	3.85%	0.60-1.80%
HIV-1 Antibody	Non-reactive	Non-reactive indicates no detectable antigen/antibody
HIV-2 Antibody	Non-reactive	Non-reactive indicates no detectable antigen/antibody
HIV 1/2 Antigen/Antibody	Non-reactive	Non-reactive indicates no detectable antigen/antibody

**Table 3:** Absolute Neutrophil Count (ANC) Chronological Trend vs. Antibiotic Regimen Summary of absolute neutrophil count (ANC) and neutrophil % for the patient since the initiation of antibiotic therapy. Day 1 - initiation of oral vancomycin tablets; day 5 - admission to hospital and switch to vancomycin solution; day 11 - change in regimen to oral fidaxomicin. ANC Reference Range: 1.70 - 7.00 x 10^9^/L QID: Four times per day; BID: Twice per day.

Days Hospitalized	WBC Count (10^9/L)	ANC (10^9/L)	Neutrophil %	Medication	Dosage
N/A	6.40	5.50	85.80	Oral Vancomycin Tablets	125 mg QID
1	3.37	1.76	52.20	Oral Vancomycin Solution	125 mg QID
2	2.26	1.20	53.10	Oral Vancomycin Solution	125 mg QID
3	2.10	0.77	36.60	Oral Vancomycin Solution	125 mg QID
4	3.20	1.31	40.90	Oral Vancomycin Solution	125 mg QID
5	2.71	1.03	38.10	Oral Vancomycin Solution/Oral Fidaxomicin	125 mg QID/200 mg BID
6	2.78	1.39	50.00	Oral Fidaxomicin	200 mg BID
7	3.08	1.53	49.70	Oral Fidaxomicin	200 mg BID
8	3.12	1.52	48.80	Oral Fidaxomicin	200 mg BID
9	3.67	1.68	45.70	Oral Fidaxomicin	200 mg BID
10	4.34	2.13	49.10	Oral Fidaxomicin	200 mg BID
11	4.98	2.61	52.40	Oral Fidaxomicin	200 mg BID
12	4.68	2.30	49.20	Oral Fidaxomicin	200 mg BID

Additional inpatient medications, such as Amlodipine 10 mg daily, Carvedilol 3.125 mg twice per day, and Pantoprazole 40 mg twice per day, were deemed as unlikely causes. Reticulocyte count was elevated, suggesting intact marrow function. HIV testing was negative.

On hospital day 5 (day 11 of vancomycin therapy), due to ongoing diarrhea and concern for antibiotic induced-agranulocytosis, vancomycin was discontinued and replaced with oral fidaxomicin, with plans for a 7-day course. The Infectious Disease team was consulted and reiterated our suspicion for C. difficile diarrhea with vancomycin-induced leukopenia.

**Figure 1:**
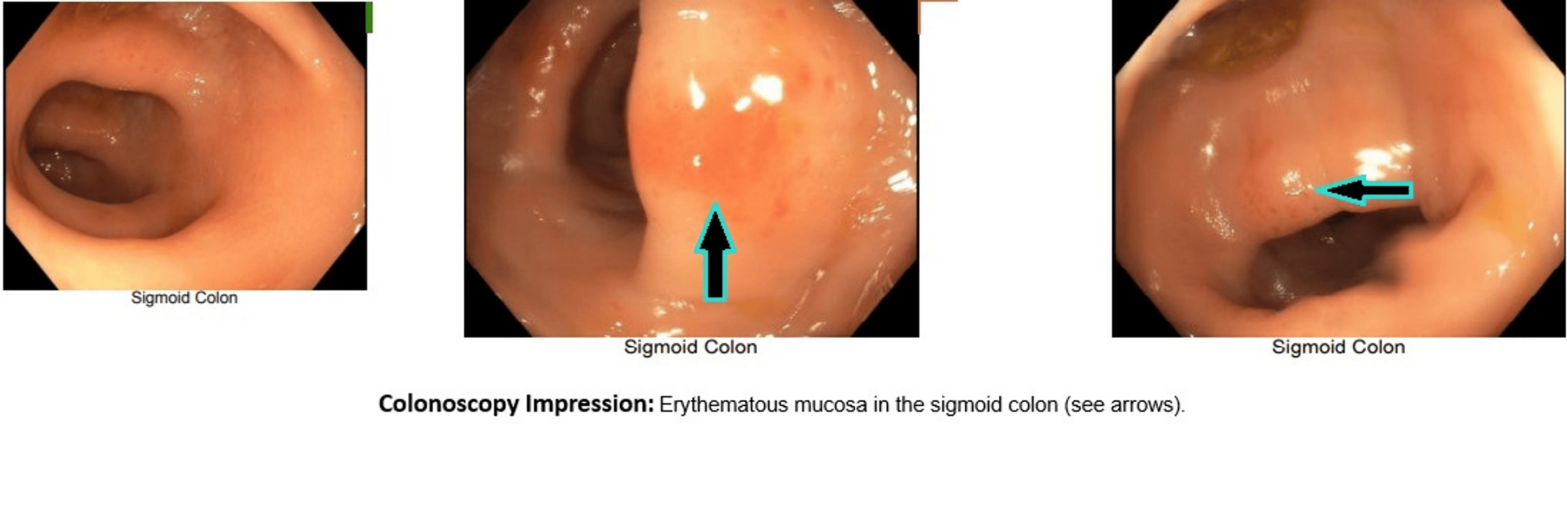
Colonoscopy Findings

A colonoscopy on hospital day 7 revealed mild erythema in the sigmoid colon (see Figure 1). Biopsies showed mild chronic inflammation and focal cryptitis, with no evidence of malignancy, microscopic colitis, or granulomas.

**Figure 2:**
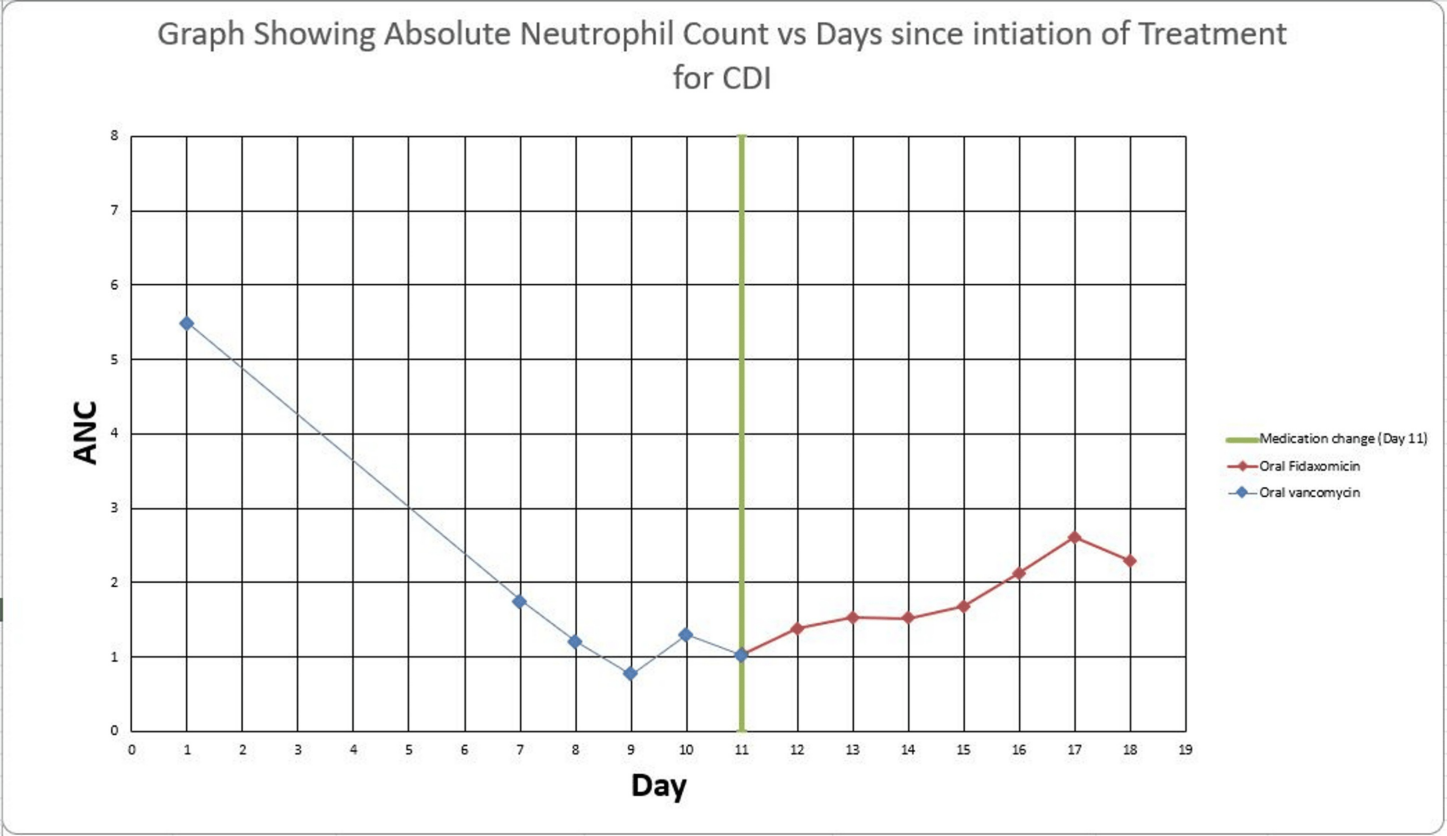
Graph showing Absolute Neutrophil Count (ANC) vs Days since Initiation of Treatment for CDI Summary of absolute neutrophil count (ANC) for the patient since the initiation of antibiotic therapy for Clostridioides Difficile Infection (CDI). Day 1 - initiation of oral vancomycin tablets; day 5 - admission to hospital and switch to vancomycin solution; day  11 - change in regimen to oral fidaxomicin (green stripe).

Following the switch to fidaxomicin, on hospital day 7 (two days after the discontinuation of vancomycin and initiation of fidaxomicin), the patient’s ANC improved to 1.53 and normalized to 2.13 by hospital day 10 (Figure 2). Her diarrhea also resolved. At discharge, her ANC was 2.30 and bowel movements were formed. The clinical decision to switch to fidaxomicin proved effective, both in resolving C. difficile diarrhea and in reversing the hematologic adverse effect.

## Discussion

Systemic absorption of oral vancomycin can increase in patients with certain risk factors, such as renal insufficiency, severe CDI, high doses (e.g., >500 mg/day), prolonged therapy (>10 days), intensive care unit admission, and gastrointestinal inflammation [2, 12]. In our case presentation, the patient developed agranulocytosis temporarily related to oral vancomycin solution, with a clear decline in ANC following initiation and subsequent recovery after drug discontinuation. Other medications administered, such as amlodipine, pantoprazole, and carvedilol, are not commonly associated with agranulocytosis, making vancomycin the most plausible culprit. The elevated reticulocyte count further supported preserved bone marrow function, pointing toward a drug-induced peripheral neutrophil destruction mechanism rather than marrow suppression.

Agranulocytosis, characterized by a severe reduction in granulocytes (specifically, an absolute neutrophil count below 500/mm³), is a rare but serious hematologic adverse effect associated with vancomycin use [13-15]. The incidence of reversible neutropenia due to vancomycin therapy is estimated to be up to 8% in hospitalized patients [2]. Previous studies have failed to recognize a strong association between the total daily dose or serum concentration of vancomycin and the progression of agranulocytosis, which makes it difficult to envisage the likelihood of incidence [11, 16]. Early identification through routine blood analysis and prompt discontinuation of the antibiotic are crucial for managing such adverse events; by extension, this applies to any medication with a narrow therapeutic index and notable interaction regardless of the route of administration [15]. If vancomycin-induced agranulocytosis or neutropenia is suspected, prompt discontinuation of the antibiotic is key, often leading to neutrophil count normalization within days [13,17].

Previously, direct bone marrow toxicity was a postulated mechanism of vancomycin-related agranulocytosis. However, swift recovery of neutrophils after the cessation of the drug or the administration of granulocyte colony-stimulating factor (G-CSF) indicates that myeloid bone marrow precursors remain intact [14, 15]. Bone marrow examinations in suspected cases have revealed normal myeloid precursors, thereby refuting direct marrow toxicity [15]. These data suggest a reversible injury or peripheral destruction as opposed to permanent destruction of the marrow [14].

Antineutrophil cytoplasm antibodies (ANCA) have been identified in conjunction with neutropenia, indicating their potential role in neutrophil lysis via complement activation [14]. Drug metabolites could possibly function as haptens, attaching to neutrophil membranes, inducing antibody production, which results in complement-dependent cytotoxicity [15]. This process typically occurs after at least 12 days of IV vancomycin therapy, with a considerable number of cases arising between 20 and 28 days [13-15]. Research shows that neutropenia is linked to treatments that last longer than 7 days, with most cases showing up after day 20 [12]. Extended treatment or re-exposure can also lead to a more rapid onset, with incidence in hospitalized patients is estimated at 2-8% [11, 13, 17, 18]. An ANCA test can, however, be expensive and would not typically be performed on a patient treated with oral vancomycin.

Limitations

Potential limitations of our case include the lack of stool culture, ANCA, serum vancomycin, and broader infectious testing/workup. These studies are not included in the standard of care, thus were not performed. Although our patient was not screened for inherited causes of neutropenia (i.e., Duffy-null), it felt unnecessary given her previously normal ANC values. Broader infectious testing (i.e., COVID, Epstein-Barr Virus) felt superfluous due to presenting symptoms in addition to the observed ANC improvement upon antibiotic change.

Future investigations are necessary to ascertain any potential dose-dependent relationship between vancomycin and drug-induced agranulocytosis. Concurrently, additional studies can also explore the utility of ANCA screening in patients with medication-mediated neutropenia. Lastly, for patients with treatment-refractory diarrhea such as our own, stool cultures may be warranted to further evaluate for reduced vancomycin susceptibility.

## Conclusions

To our knowledge, this case represents the first documented case of vancomycin oral solution-induced agranulocytosis. Furthermore, it highlights three important clinical considerations. First, clinicians must remain vigilant for hematologic toxicity in patients receiving vancomycin, regardless of the route of administration. Second, monitoring ANC should be considered in patients receiving prolonged therapy, particularly those with gastrointestinal pathology that may increase systemic absorption. Third, early recognition and prompt discontinuation of vancomycin, with substitution to an alternative therapy such as fidaxomicin, can result in resolution of both infectious and hematologic complications. Recognition of this adverse drug reaction is important, as delayed diagnosis may place patients at risk for severe infection, sepsis, and poor outcomes.
